# Physical Literacy and Physical Activity in Early Years Education: What’s Known, What’s Done, and What’s Needed?

**DOI:** 10.3390/children11111355

**Published:** 2024-11-08

**Authors:** Natalie Weir, Andy Pringle, Clare M. P. Roscoe

**Affiliations:** Clinical Exercise Rehabilitation Research Centre, School of Sport and Exercise Science, University of Derby, Derby DE22 1GB, UKc.roscoe@derby.ac.uk (C.M.P.R.)

**Keywords:** physical literacy, physical activity, early years education, physical development, early childhood, intervention

## Abstract

**Background:** Physical literacy (PL) is increasingly recognised as essential for fostering lifelong engagement in physical activity (PA), particularly when nurtured in early childhood. Yet there remains limited understanding of how stakeholders in early years (EY) education perceive, value, and implement a PL-informed approach. This study aims to explore knowledge and beliefs regarding PL and PA in relation to 3–5-year-olds, investigating key questions around perceived importance, current practices, and barriers to implementation. **Methods:** A concurrent mixed-methods approach was used, incorporating semi-structured expert interviews (*n* = 11), focus groups (*n* = 22), and a survey (*n* = 210). Thematic analysis was used to identify key themes from qualitative data, and survey data were analysed to complement and triangulate the qualitative findings. **Results:** The findings revealed variation in stakeholders’ awareness of PA recommendations and confusion over terminology. Whilst stakeholders acknowledged the importance of PL, there remains uncertainty about the connection between theory and practical application. Identified barriers included resource limitations, conflicting priorities, and insufficient training and policy support. Feedback on an educational PL-EY model was generally positive, suggesting strong potential as a tool to support PL understanding and application in early childhood contexts. **Conclusions:** Given the pivotal role of EY education in shaping children’s behaviours, health, and wellbeing, this study highlights the necessity of a holistic approach to interventions, strong stakeholder involvement, and evidence-based practices to foster PL in EY children. The PL-EY model presents a promising direction for future resources and education and raises critical questions about what effective interventions to develop PL in this age group should consider and look like.

## 1. Introduction

All children, regardless of their background or circumstances, should be supported in developing positive relationships with physical activity (PA) from the earliest opportunity. The World Health Organization (WHO) defines PA as any voluntary bodily movement produced by skeletal muscles that requires energy expenditure [1]. The UK Chief Medical Officer’s PA guidelines for children under five years recommend at least 180 min of PA per day, including 60 min of moderate-to-vigorous physical activity (MVPA) [2]. However, less than 10% of 2-to 4-year-olds meet this target [3], and 1 in 5 children in the UK are overweight or obese by the age of five years [4]. The impact of childhood obesity on the NHS is significant, costing £6.1 billion annually, with predictions suggesting this will rise to £10 billion by 2050 [5]. This trend underscores the urgency of addressing PA levels in early childhood.

Preschool children engage in PA of varying intensities—from light-intensity movement (slow walking) to MVPA such as running and jumping. Evidence consistently shows that PA in early childhood can improve cognition and mental and physical health, and reduce the risk of future obesity [6,7]. The holistic concept of physical literacy (PL) provides a powerful lens for examining movement in relation to PA and motor skill outcomes [8]. It is described as an individual’s relationship with movement and PA throughout life (representing a deeply personal, holistic, lifelong, and constantly evolving concept more than merely acquiring the capabilities for PA), encompassing the cognitive, affective, physical, and social domains of learning [9]. PL incorporates physical competence, confidence, and motivation for PA [10] and acknowledges the importance of engaging in different types of movement opportunities for a positive and meaningful relationship with PA. A PL-informed approach is increasingly seen as a critical factor in promoting PA and supporting children’s physical development. Moreover, a positive correlation between PL and PA levels in young children is also starting to be demonstrated, whereby higher PL is associated with increased PA and reduced sedentary behaviour in preschool-aged children [11,12], particularly in relation to the domains of physical competence, motivation, and confidence [13]. Children with greater PL are also considered more likely to meet daily PA guidelines [14]. A positive moderate association between PL and physical and psychosocial wellbeing has also been shown [15].

Low levels of PA and poor PL in young children can have negative physical and mental health consequences [16] and sadly, physical and health inequalities have worsened since the COVID-19 pandemic [17]. A marked increase in sedentary behaviours and negative attitudes towards PA [18] has significant implications for early childhood development, contributing to delayed motor development as well as wider developmental repercussions such as diminished cognitive and social skills [19]. In response, PL is gaining attention and is increasingly incorporated into national policy documents supporting the need for children to be more active. However, the absence of a curriculum for physical education (PE) in the Early Years Foundation Stage (EYFS) prior to Key Stage 1 (the phase of primary education for pupils aged 5 to 7 years in England) as outlined by the national curriculum [20] leaves EY educators to navigate this critical developmental stage often with insufficient training, resources, or guidance in relation to PA [21].

In addition, much of the work in exploring childhood PL has focused on children above the age of 5 years, which has left a gap in the preschool years [22], and little is known about EY educators’ perceptions of PL and its role in PA. Whilst physical development (PD) is one of the three prime areas of learning and development in the EYFS Statutory Framework (that sets the standards for the learning, development, and care of children from birth to 5 years), there is little insight into how PA connects to the broader components of PL. This is how a child moves, connects, thinks, and feels during movement opportunities (representing physical, social, cognitive, and affective areas of learning and development, respectively). Published content on transforming the conceptual ideas of PL into interventions has grown considerably in recent years [23]. A recent review of PL interventions demonstrated that the majority of scientific evidence on PL interventions has been delivered by projects from Australia, Canada, and Great Britain, however most interventions include goals or content related to physical competence, with other domains often not sufficiently addressed [24]. Similarly, interventions explored in relation to early childhood are limited [25] and mainly explore theory-based education and delivery in either a childcare or parental context. This gap in understanding presents an opportunity to explore how PL is valued and applied in EY settings alongside the practicalities, challenges, and opportunities for delivering interventions aimed at fostering PL. To the authors’ best knowledge, there has been limited research examining the perceptions and understanding of the relationship between PL and PA in the EY, which is a vital first step to better comprehend its position within EY education and practice. This study aims to address this research gap by seeking to understand how the concept of PL is understood, applied, and valued by a variety of EY stakeholders, such as educators, parents, and policy makers. In addition, gathering feedback on the feasibility of measuring PA and PL levels of 3–5-year-old children is explored alongside identifying potential future intervention design key themes, opportunities, and challenges.

## 2. Materials and Methods

### 2.1. Study Design

This study employed an interpretivist study design, which seeks to explore the perceptions of individuals within their unique social contexts [26]. Interpretivism was deemed an appropriate lens for this research, as it allowed for the examination of understanding and experiences [27] in relation to PA and PL development in education for 3–5-year-old children. A concurrent mixed-methods embedded design was used, effectively combining rich in-depth qualitative insights with numerical data, offering a broader understanding of the research problem [28]. The two methods can be conducted simultaneously [29], and, in this study, qualitative data (semi-structured focus groups and one-to-one expert interviews) provide the primary source of insight, supported by the quantitative data (survey via the Qualtrics platform). Whilst the interview insight was prioritised, the survey allowed for a wider geographical perspective and, collectively, insights from a wide variety of professionals and stakeholders were captured (from parents, educators, and managers to those with a specialised interest or expertise in early childhood development). This approach ensured that the data collection methods were triangulated [30], enabling a more robust analysis of the research questions and facilitating a deeper understanding of PA and PL in the context of the EYFS.

### 2.2. Participants

The inclusion criteria required that participants were either parents of children aged 3–5 years old, professionals who worked directly with children of this age, or individuals with knowledge and expertise related to PA or PL in the EY. All participants were required to provide informed consent before their participation. Participants were recruited through a combination of direct contact with relevant individuals and stakeholder groups as well as through social media channels inviting responses to an open call. Snowball sampling was also utilised by encouraging participants to invite colleagues, friends, or family members who met the study’s inclusion criteria. Participants were invited to take part in one of three data collection methods (survey, focus group, or expert interview). Interested individuals contacted the researcher via email to confirm their participation and arrange a suitable time and date for the interview. A Microsoft Form was used to streamline scheduling for focus group participants, offering them multiple time and date options for participation.

The final sample was composed of a heterogeneous set of participants from diverse geographical and professional backgrounds across 11 expert interviewees (*n* = 11), 4 focus groups (*n* = 22), and a survey (*n* = 210). Expert interview participants were made up of 73% female (*n* = 8), from locations in England (*n* = 10) and Wales (*n* = 1). They either taught children directly (*n* = 5) or held nursery managerial positions (*n* = 2), academic (*n* = 1), or policy and programme roles within support organisations (*n* = 3). The focus group interviewees were similar: they were 73% female (*n* = 16), and included parents (*n* = 4), practitioners (*n* = 7), senior leaders (*n* = 2), activity providers and community organisation stakeholders (*n* = 5), or a combination of roles (*n* = 4). The majority were from locations in England (*n* = 21) with a wide geographical spread including Blackpool, Cheshire, Greater Manchester, Shropshire, Derbyshire, Suffolk, and Plymouth.

Survey participants were predominantly parents (58%, *n* = 121) or practitioners (40%, *n* = 84). Other respondent roles included childminders, sports coaches, activity providers or those who worked in health, community, or charitable organisations. Some 18% (*n* = 37) were parents with another applicable role. Most participants were female (83%, *n* = 175). A diverse age distribution between 18 and 55+ years was represented with 35–44 years (40%, *n* = 83) being the most common. Most respondents worked or had children in state nurseries or primary schools (52%, *n* = 108) or private, voluntary, or independent settings (29% *n* = 60); others were childminders, homecare, or children’s centre, play, or holiday club settings. Participant responses represented children’s attendance in education settings across 39 English counties (*n* = 170) out of a possible 48, with 29% coming from the researcher’s home county of Derbyshire (*n* = 61). Responses from 13 other countries (*n* = 20), including the 3 UK home countries of NI, Wales, and Scotland, were also included.

### 2.3. Ethical Considerations and Consent

Ethical approval for the study was obtained from the University of Derby’s College of Science and Engineering Research Ethics Committee (ETH2223-4776). Consent was gathered via the Qualtrics platform for survey respondents. Interviewees were provided with an online link to complete their consent through a Microsoft Form. Consent forms covered aspects such as the purpose of the study, potential benefits and risks, confidentiality, and the use of recorded data. All the data, including video recordings, transcriptions, and survey responses, were securely stored on the university-approved Office 365 platform. Access to any identifiable data was restricted to the lead researcher to maintain confidentiality. Interviewees were also provided with a link to the online meeting (via Microsoft Teams) and a copy of the PL-EY model poster. Participants were informed about the data collection methods, the need for video and audio recording, how their data would be used, and their rights to withdraw from the study at any time without consequence verbally at the start of the interview or focus group.

### 2.4. Data Collection

To provide structure and direction to the research while allowing room for unexpected insights to emerge, semi-structured interview schedules were devised to allow flexibility while addressing our research aims—that is, to solicit information regarding the understanding, value, and application of PL and PA in relation to 3–5-year-olds. Questions were formed in relation to 6 a-priori themes, an effective practice involving the creation of predetermined categories based on the research question and shaped by the literature or theory [31]. These 6 themes guided the development of the interview and survey questions based on gaps and themes identified in the literature [32]. The interview scripts and survey questions can be viewed in Appendix B.

A total of 11 expert interviews were conducted on a one-to-one basis over a two-week period from 21 November to 5 December 2023, according to participants’ availability. The semi-structured focus groups were held over a 12-day period from 27 November to 8 December 2023. Four sessions were conducted, each lasting between 44 and 73 min, in accordance with the maximum two hours stated as good practice [33], and with a range of four to seven participants. Sessions were held at varying times (weekday lunchtimes and evenings) to inclusively accommodate a variety of participants’ schedules. All the interviews and focus groups were recorded and transcribed, allowing for comprehensive data analysis. Following the completion of the interviews and focus groups, participants were thanked for their time and followed up with a thank you email.

The survey was designed to capture both quantitative and qualitative elements of PA and PL understanding among EY educators, parents, and other stakeholders. To ensure reliability and consistency, the questions were built similarly to previous research that used scales to assess the understanding of PL [34,35], using 5-point Likert-scale questions to assess knowledge and familiarity (e.g., “How familiar are you with the concept of PL? 1—Not familiar at all, 5—Very familiar”). Open-ended questions invited participants to elaborate on barriers, opportunities, and key considerations for intervention design. During the survey development, we prioritised accessibility, stakeholder relevance, and the use of clear terminology with definitions to enhance understanding and avoid confusion around concepts. The survey remained open for responses between 13 November 2023 and 17 January 2024, enabling a broad range of participants to contribute to this mixed-methods data set.

### 2.5. Data Analysis

**Figure 1:**
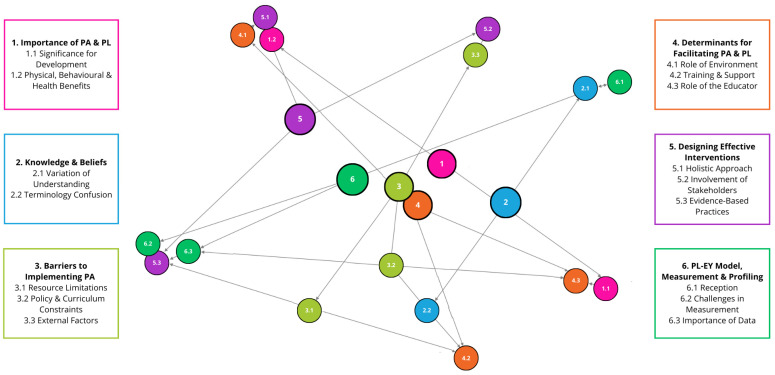
Thematic map diagram.

Microsoft Excel was used to summarise the demographic data and the 334 survey respondents’ data. The survey had a 63% completion rate (*n* = 210) with incomplete data excluded from the analysis. Expert interview data (*n* = 11) and focus group data (*n* = 22) were examined and coded according to Braun and Clarke’s [36] six-phase thematic analysis model, using a critical reflective approach. Involving familiarisation of the data, the generation of initial codes, the identification of themes, the review and refinement of themes, defining and naming themes, and producing the final report—this data analysis process has been commonly used in PA and PL research [24,37,38]. The thematic analysis focused on capturing recurring themes and patterns within participants’ responses related to PA and PL understanding and intervention design. Following the transcription and checking and reading of the interview transcripts through to saturation, coding was used to identify and group interesting features in the data. The original 6 a-priori themes created were used to group codes into coherent themes, but to encapsulate broader concepts, 8 themes and 20 sub-themes were generated. The themes were refined, reduced, and sense-checked by the research team to confirm credibility and trustworthiness. This ensured that different perspectives and interpretations of the data generated from participant interviews were thoroughly explored and discussed, contributing to the development of a theoretically robust argument [39]. A final set of 6 themes (and 16 sub-themes) were finalised in a team discussion to better represent the research aim and group compelling data extracts. These themes were used to structure the results and present a cohesive narrative around the knowledge, importance, and implementation of PA and PL development in 3–5-year-olds in education. While we present and discuss the themes individually, we recognise their interconnectedness for building a novel contribution to understanding, and the themes and how they relate to each other are illustrated in Figure 1.

## 3. Results and Discussion

The results are presented by using a selection of extracts within the text and are summarised in Table 1. Key discussion points and a summary for each of the 6 theme sections are provided alongside the results. Quotes are referenced using codes that were created for each participant, e.g., EE1 (EY educator, participant 1), EA6 (EY academic, participant 6), and EEM9 (EY nursery manager, participant 9), or include numbers if they were from a focus group e.g., F4P1 (female parent from focus group 4). Survey participants are labelled by number, e.g., P21 (21st survey response). A participant coding key can be viewed in Appendix A (Table A1).

### 3.1. Importance of Physical Activity and Physical Literacy

EE1 states, “PA and movement are fundamental to everything else. They are crucial and fundamental to every child’s development and high-level learning happens through play”. The benefits of PA extending beyond PD to encompass behavioural and health outcomes were highlighted with EE3 commenting “it’s just what we are born to do. Fundamentally, our bodies are designed to move and the sedentary lifestyle that we live in does not work well with our bodies and brains, and that’s why we’re seeing an increase in mental health behaviours”. Such insights align with the increasing focus on reducing sedentary behaviours, particularly in young children, to safeguard both physical and mental health [40]. This ties to the idea that PA fosters more than just motor skills; it is integral to behavioural regulation, as noted by F4O3: “families are complaining about the children’s behaviour and saying that…they’re being destructive at home and they can’t control their behaviour—and we explore what happens if you go and give them an opportunity to be active”. Reinforcing the connection between PA and energetic play to improve self-regulation supports the increasing literature relating to young children [41] and in the EY classroom [42]. F3E1 provided a practical example: “…we saw the improvement afterwards and it was huge for behaviour, it was huge for communication, especially within the girls and preschool…it benefited educationally”. EM11 also observed the wider learning that occurs through movement and play: “They get the heart rate up but also they’re learning how to climb and learning how to socialise and take turns”. Existing research also suggests that PA and play may improve cognitive development [43] and children’s learning and academic performance [44]. The interconnected nature of PA and play is emphasised with F2PE1 describing PA as “learning through play and movement”, and F1O1 comments that we need to “allow natural physical play to take place…that are safe but obviously have got lots of risks and challenges”.

Overall, there was a recognition of the importance of a PL-informed approach by interviewees and a recognition of the early childhood years being vital for nurturing children’s wider relationship with movement. Whilst this is a natural viewpoint for those that are involved in the EY industry, it reinforces research emphasising that early experiences are significant for shaping children’s health and developmental outcomes [45]. EE2 highlighted the importance by stating “you can kind of hit the top iceberg if you want to by just introducing the term [PL], or you can get right down to the very bottom of that iceberg and you can really start to explore the real benefits of what PL is”. This was supported by the survey results which found that 75% (*n* = 158) of the respondents considered PL to be important for 3–5-year-olds (where they rated the importance at 8 or above out of 10). Interviewees’ strong corroboration of the importance of PA in early childhood mirrors the broader argument in the literature that early exposure is key to fostering long-term PL and PA engagement.

In terms of the survey (*n* = 210), where respondents were asked to rank PD outcomes of 3–5-year-olds in order of importance, ‘achieving enough PA’ only rated 7th out of 10. These results are displayed in Table 2. A Kendall’s W test to explore the level of agreement was conducted (w = 0.320, x^2^ (4) = 604, *p* = < 0.001) and indicated a moderate level of agreement among the raters and a significant difference across the related groups. There were clear trends, particularly in relation to the top 3 (where enjoyment and motivation had the highest mean ranking), aligning with findings that emphasise the importance of fun. For example, play that is perceived to be ‘fun’ tends to engage and motivate children to perform activities over a much more prolonged period of duration [46]. Gross motor skills and fine motor skills are ranked highly, above fundamental movement skills (FMS)—mirroring the current focus in EY PD policy and assessment (reflecting the two early learning goals related to PD in the EYFS) and the narrow focus in the literature on motor skill development. This is echoed in interviewees’ views, with F4O2 commenting on the importance of “movement through play for fine and gross motor skill development”. The variation in how survey participants prioritised the outcomes could reflect differences in personal experiences, professional backgrounds, and varying interpretations of what is most crucial for 3–5-year-olds—especially since the survey consisted of a mix of stakeholders, including a large proportion of parents (58%, *n* = 121).

The findings underscore the role and experiences of PA, supporting not only PD but also cognitive, behavioural, and social elements in early childhood. While interviewees and survey respondents recognised the importance of PL from an early age, differences in priority outcomes suggest a need to embrace approaches that integrate play, enjoyment, and engagement as well as FMS as critical components.

### 3.2. Knowledge and Beliefs About PA and PL

Survey responses (*n* = 210) highlighted a poor awareness of the recommended daily PA levels for 3–5-year-olds. Whilst 76% (*n* = 159) stated that ‘yes’ they were aware, only 60% (*n* = 96) identified the correct 180 min (with 60 min of MVPA). As EM07 pointed out: “Many professionals aren’t aware of the CMO guidelines…There are so many other priorities.” This lack of awareness can significantly hinder the implementation and value of adequate PA. Concerns for the variation in understanding and misconceptions about the need for structured PA in the EY are demonstrated by EE1 stating: “There is almost this belief that with children between 0–5 it [PA] just happens naturally, so we don’t need to do anything—you open the door and let them run and that’s it”. F2PE1 suggested that “in a nursery setting, when the majority of the workers have like their Level 3 in childcare, they don’t really cover the sort of the PL side and I don’t think they’d actually know the PA requirement”.

The terminology surrounding PA and PL as well as PD and PE often causes confusion, as EE3 pointed out: “there are so many interchangeable terms that I think there needs to be something for educators to really comprehend and understand the PD process”. The existing literature [47] points out that PL is often incorrectly defined or assessed by the ability to perform fundamental movement skills (FMS) when it is a multidimensional construct [48], and this was echoed in the differences in understanding demonstrated by our interviewees. When asked to verbalise what they understood PL to be, some participants were able to illustrate good knowledge. EE3 mentioned “competence, motivation, confidence and enjoyment” as important strands. However, those that were more general educators or parents lacked this knowledge, with F1M1 commenting that “it’s the understanding of the physicality of actions and the competence or developing the competence” and EA6 observing that “PL, then to me, is not just the PA…it’s also whether you’ve got the abilities to move, so the FMS” and EE4 stating “like the knowledge of how to run and change direction and balance and again I guess the competence”. Similarly, F4PE1 commented: “I think FMS, that’s kind of my go to”. Whether the phrase PL itself acts as a potential barrier for adoption by EY educators was raised. EM11 questioned “when you say that word [PL], it’s like ohh, it’s got to be in relation to reading” and ED10 observed that “it sounds academic”. Some 40% (*n* = 74) of the survey respondents stated they were extremely familiar or very familiar with the term PL, but as a definition was not initially provided it was not possible to check or challenge this knowledge in terms of the accuracy or misrepresentation of the term. These perspectives highlight the need for clearer communication and education around the concepts of PL and its purpose in EY children’s development. The results support existing evidence that suggests there are notable gaps in the knowledge and misconception of PL [49] and practical skills for developing PL, particularly within early childhood education programmes [25], that require further action.

The findings reveal gaps in the awareness and understanding of PA guidelines and PL in practice among EY stakeholders, compounded by confusion around the terminology and misconceptions about structured versus unstructured PA needs for young children.

### 3.3. Barriers to Implementing PA Guidelines and Developing PL

If stakeholders do not understand PL (as holistically incorporating the four domains: physical, cognitive, social, and emotional), then they cannot effectively support a PL-informed approach in practice. However, the considerable learning and developmental outcomes required in the EYFS were considered a challenge, with resource limitations and lack of training coming across as significant perceived barriers. For example, EE4 highlights: “Practitioners get bombarded with posts about everything, like safeguarding and anything else like additional needs”. EE1 emphasised, “accessible training is really important, and it needs to come from evidence-based specialists” and EM11 stressed the need for “[resources] that educate and support professionals and parents and caregivers on the link between PL and PA so that they can understand”.

Policy and curriculum constraints also pose challenges for implementing PA guidelines, as EE2 noted: “there’s no talk about PL in EY. There’s no policy. There’s no documentation, and there’s nothing that says anything about what PA actually looks like in EY”. These broader systemic issues are also highlighted by ED10, who suggests that “getting the Department of Health and Department for Education to talk about anything together is challenging” and that “the education side needs to recognise that the evidence being presented by the health side is not a pretty picture and needs to be actively addressed”. Observations about the lack of coherence between health and education policy makers resonate with broader critiques in the literature about the siloed approach to child health and education [50], suggesting the need for better interdepartmental awareness and collaboration between the health and education sector to streamline policy initiatives and widen the narrow narrative around PD in the EY to include more content around PA and PL. This requires intervention and action; this is especially opportunistic at the time of writing as in the UK, the current political administration is entering its first months of governance. Similarly, P5 suggests there is a “lack of understanding of the value of PA by senior leaders” and F1E1 emphasises that at the practice level, “we need to value that [PA and PL] and have a whole team approach and a whole school approach”. These observations underscore the need for policy-level changes to support embedding PA recommendations and adopt a PL-informed approach in EY settings. Notably, researchers and policymakers are increasingly using the concept of PL to scaffold interventions targeting increasing children’s PA [51,52] but this is less evident in application to early years environments.

All the participants across the focus groups and expert interviews believe the current PA recommendations should be achievable, but only 17% (*n* = 25) of the survey participants believe there is “always” an adequate opportunity for children aged 3–5 years old to reach PA recommendations, with 71% (*n* = 105) believing there is “sometimes” or “often” an adequate opportunity. The challenge of inactive EY educators was also highlighted. For example, EE4 points out: “the other barrier I see is a lot of practitioners, and especially the older ones, don’t do much PA themselves.” P46 contributes that “teaching styles require children to be seated/still throughout long periods of time while learning” and EE3 reinforces this by suggesting that “everybody says our curriculum just does not allow time”. This lack of personal engagement with PA and attitudes towards movement can influence how educators approach it in their practice. However, external factors, including parental attitudes and socioeconomic constraints, are considered further barriers. For instance, EM11 observed that “parents were either very supportive or had very different views on what was appropriate for their children” as well as noting environmental challenges: “The challenge is that [settings] are all different, so some don’t have outside space, some have a very small outside space”. P27 noted differences in “access to outdoor space” and P22 questioned if the weather could significantly “limit outdoor play”. Therefore, there are a myriad of variables that have an impact on the likelihood of children in this age group being sufficiently active, as EE2 sums up by stating: “It depends on the environment, the people around them, the situation that that child is in, whether they can achieve it [PA recommendations] or not”. EM07 highlights that “every setting is so different…some settings do discrete PE sessions; some have a real emphasis on everyone moving”. F2M1 comments that there should be more opportunities for movement in the EYFS as “they’re not sat at tables yet…and are able to explore their own physicality in a completely different way to an older child”. However, P121 summarises that “the EYFS framework and school policies formalising the EY has had a significant role in reducing play experiences in the EY classroom”.

In summary, barriers include resource limitations, lack of training, and restrictive policies. Variability in settings, educator attitudes, and external factors like parental attitudes and environmental constraints further limit PA opportunities for young children. These results suggest a pressing need for policy adjustments, interdepartmental and stakeholder collaboration, and a more holistic, supportive approach to embedding PL-informed practice effectively across diverse EY environments.

### 3.4. Determinants for Facilitating PA & PL

Physical environments and educator attitudes equally play a huge role in facilitating PA, as EE3 comments: “if it’s an environment where it’s small world play and children are sitting on a mat or looking at circle time, then that’s going to be very different levels of PA, mainly sedentary behaviour. So, this [environment and educators] will dictate that”. The literature reinforces this, suggesting that nursery and preschool environments are often organised so that boys, older children, and highly active children benefit more compared to girls, younger pre-schoolers, and children with lower MVPA levels [53]. Research has also shown that in about 50% of classrooms, children do not have sufficient opportunities to be physically active during the school day [54]. Indeed, EE1 emphasises the importance of intentional planning and the need for “planning the curriculum to ensure there are those key moments in the day for PA to happen.” These insights highlight the need for thoughtful environmental design and curriculum planning in EY settings and EM11 observes that the “focus tends not to be on the PA. It tends to be on speech and language and things like that…[the way they] traditionally delivered [it] doesn’t include PA”. This need for adequate training and support for educators emerged as a key determinant for facilitating PA and a PL-informed approach and EM07 noted that “…to provide top-quality training for our educators, whether they’re childminders or volunteers at a playgroup or educators within a setting”. ED10 suggested a practical approach: “Notwithstanding the fact that the policy isn’t going to change from the top, it may well be fertile ground to work with individual setting managers and their staff teams.” This highlights the potential for interventions that support change in the absence of top-down policy shifts. Survey respondent P27 commented that we “need to have access to reliable PL research and resources with a practical application to EY and it needs to be embedded into all EY training”.

The role of the educator is widely demonstrated by interviewees to be critical in facilitating PA, as EE2 states: “you have to be like the facilitators for the children to be able to hit those [PA] guidelines”. EE1 also commented that “a role model is probably one of the most crucial things you can ever have within a setting.” A good example in practice was provided by EE4: “If the staff go out in the garden and just lean against a tree or lean against a fence and they are sedentary, what are the children going to learn?”. Survey respondent P118 states the need for “responsive adults to model and support movement. Allow it to be child-led—adults to follow child’s lead” and F3P1 highlights the differences that arise in values and options when “exercise is this chore…or where it’s all about fun and playing”. These perspectives underscore the active role educators must play in promoting PA as well as the most holistic components that play a part in a child’s ability to be physically active, such as motivation: P132 notes that “children have poor PL and don’t join in and are not encouraged”. Our findings also emphasise the need to explore how we can better educate and engage with early education stakeholders, and one such theory that has been explored in relation to EY educators is the Capability, Opportunity, Motivation-Behaviour (COM-B) model [55]. This model highlights the complex nature of the role of the educator and how their own engagement with PA can either facilitate or hinder children’s PA. It has also been suggested that pedagogical experience from an educational background other than PE is an obstacle to understanding PL [56] comprehensively, reinforcing the need for intervention development in this area. The long-term implications may be that if early childhood education and physical education work together, they will be able to inspire more meaningful experiences in teaching physical activities that contribute to the formation of children’s PL.

Effective facilitation of PA and PL development in EY provision requires a supportive physical environment, intentional curriculum planning, and educators who actively model and encourage movement. The findings emphasise the value of providing educators with accessible, evidence-based training, communication, and resources relevant to EY children.

### 3.5. Designing Effective Interventions

There is a rapidly evolving evidence base for interventions seeking to develop PL, particularly in children [57]. Effective interventions for promoting PA and developing PL require a holistic approach, which considers multi-level determinants, and the variety of contexts, environments, and support systems, as EE5 emphasises: “PL can’t be delivered. All we want is PL-informed practice. That’s what we need to go after”. The need for education, training, and comprehensive understanding is a recurring narrative, with EE3 stating that “we need to break that chain of behaviours and perspectives” and ED10 adding that “there was a period of time when [OFSTED] inspectors had commented that outdoors PA was a chance for the children to not be learning”. EM11 also comments on the need, across policy and practice, to provide “support at the earliest stage [to understand PL].” Involving various stakeholders is crucial for designing effective interventions, as EE1 noted: “it has to come from the setting and the leader of the setting”.

Within the role of PA in children’s healthy development, school-based interventions are often seen as a priority [58] and the importance of basing interventions on solid evidence was frequently mentioned with EE5 stating, “we just keep being pushed back all the time in terms of ‘What’s your evidence?’ I think any type of data strengthens that… it becomes helpful because you’ve got something concrete”. They conclude, in relation to the need for intervention, that “every time that we have an intervention…the case for change is that the status quo isn’t an option”. EE1 echoes the need for “evidence-based research to say how important this is” and F1E1 comments that “we need to get back to basics. Really big issues, they’re things that could have been solved if they had more opportunity for that freedom to move”. A recent systematic review of interventions involving parent engagement to improve positive PA behaviours and FMS found that interventions that involved parents in childcare-based settings with teacher-led instruction, were effective at improving the children’s FMS, as were studies that were parent-led and within the home environment [59]. These findings emphasise the need for more data-driven decision-making that integrates the views of key stakeholders, parents, and child-led data and raises important questions as to what effective, holistic interventions to promote PL should consider and look like in the EY.

There are 3 ‘prime areas’ of learning and development in the EYFS [60], and survey respondents were asked where they consider PL to best sit. A fourth option was given in addition to communication and language, PD and personal, social, and emotional development. This was a ‘life curriculum’—an overarching concept not limited to a single development area but which influences and is influenced by them all. Interestingly, the overarching life curriculum was considered above PD (46% (*n* = 83) compared to 34% (*n* = 61), respectively). This provides support for the potential development of PA and PL education to sit holistically across EY practice, reinforcing the interconnected nature of PA, PL and childhood development, and offers insight into how to frame intervention development and delivery. A significant step in this area would be the design and implementation of interventions based on the concepts of PL, as a notable gap is understanding PL and assessing its impact on PA levels and whether this relationship contributes to better health indicators [61].

The results support the opportunity for the integration of PL principles across EY practice, potentially framing PL as an overarching ‘life curriculum’ to embed it into all aspects of development. Designing effective interventions to promote PA and PL in EY contexts requires an evidence-based approach that actively involves a wide range of stakeholders that value positioning EY education as a foundational phase for establishing lifelong PL and health behaviours.

### 3.6. The PL-EY Model, Measurement, and Profiling

**Figure 2:**
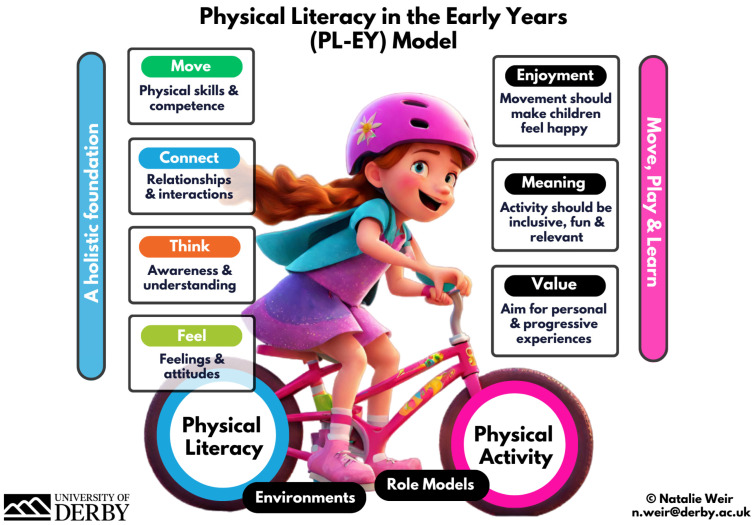
PL-EY Model.

Our results support the existing literature about how vital it is to promote healthy development in young children through a PL lens and therefore it was useful to determine if a PL in the EY (PL-EY) model was useful as an educational resource. ED10 commented that a resource like this would be well-received as long as “it is contextualised in the [education] discussion” with EE1 observing, “I think the way the [PL-EY] model has been put together is non-threatening and cleverly done”. EM07 noted its potential from a data-led perspective: “It’s important that we benchmark how many children are accessing PA and having their required 180 min of PA and of where they are in their PL journey so that we can take positive action for those children”. Constructive feedback provided on colours, text, fonts, and visualisation was used to develop a final version, as displayed in Figure 2. In the survey responses, 94% (*n* = 172) found the PL-EY model poster moderately to extremely useful in educating them and that it supports the qualitative suggestion for evidence-based, easy-to-understand resources for educators, as well as the wider community (parents, activity providers, policymakers). F2P1 commented that, prior to seeing the PL-EY model, they “equated it [PL] with motor skills, but now I see how the other elements seem to come into it”.

The feasibility of measuring PA and PL was explored and discussed in relation to challenges and opportunities and as EE2 noted, “the amount of time a child is active across their day is so hard to determine”. The need for objective data for this age group was highlighted by EM11 – “You can’t ask the five-year-old whether they’ve been doing 3 h of PA a day” – and EE4 illustrated the complexity of measuring PL: “It’s very difficult to have the holistic view of a child sometime in that snapshot data”. Survey respondent P76 commented that “schools are already measuring a lot of things, so it would need to make sure that the administration burden of monitoring PL wouldn’t be too onerous”. F1O1 added “I’ve worked with a lot of organisations that have said we’re going to bring in a PL measurement tool and then they come up with this really complicated assessment”. This aligns with the existing literature, evidencing that despite the growing recognition of PL, research linking PL to PA in young children remains limited [62], which could be due to difficulties in benchmarking metrics of PL for younger children. P46 sums this up by stating “PL has many components, and I think it is difficult to measure all of them accurately/reliably in this age group” Despite these challenges, participants recognised the importance of data in driving improvements—ED10 stated, “data talks, doesn’t it really?… We are supposed to be in an environment where policy is based on data and what works”. EE1 commented that “we need to make sure that assessments are relevant and supporting what they do next when it comes to PA”. These findings reinforce the difficult balance required between the need for an evidence-based approach and innovative measurement approaches for this age group that are not overly complicated or time-consuming. The survey results on measurement and profiling add nuance to the qualitative findings, supporting the recognition of the importance of data while acknowledging the challenges in measurement, whereby 68% (*n* = 117) think it would be “definitely” or “probably” useful to measure and profile PA levels in 3–5-year-olds with 63% (*n* = 109) thinking it would be “definitely” or “probably” useful to measure and profile PL. EA6 offers a balanced perspective: “you might not be able to measure all of these things, but with PL if you can go for the things that are key it is feasible and sort of conceivable that you will get a reliable measurement of the concept that you are trying to get at”. Some 80% (*n* = 137) of the survey respondents believe that data on PL and PA levels would “definitely” or “probably” influence their approach to the development of 3–5-year-old children, reinforcing the significant potential for intervention development through holistic, evidence-based interventions that involve the benchmarking of PA and PL, to provide insightful data on their relationship to a variety of EY stakeholders.

The PL-EY model has potential as a valuable educational resource, especially for illustrating the principle of benchmarking children’s PA and PL levels, though challenges in measuring and assessing PL for young children were noted. Stakeholders emphasised the need for practical, straightforward tools to support data-driven improvements without overwhelming practitioners. Overall, the findings reinforce the necessity for simple yet meaningful assessment methods, as well as broader support for the EYFS workforce to effectively integrate PA and understand what a PL-informed approach to early childhood development looks like in practice.

### 3.7. Limitations and Strengths

Whilst participation was predominantly female across the qualitative (73%, *n* = 24) and quantitative (83%, *n* = 175) research strands, this is reflective of educators in England [63]. Participant race and ethnic background would have been beneficial to consider but were not captured, and it is difficult to gauge if cross-cultural interpretations or differing policies and practice may have influenced international respondents (*n* = 40). Snowball sampling as a participant recruitment method is considered non-random, which can lead to a sample that is not representative of the population. Nonetheless, a strength of this research is the varied participant sample and the mixed-methods approach integrating wide-reaching survey results with interview data. By offering numerical support to the key themes, this demonstrates how different types of data can work together to provide a fuller picture.

Furthermore, few studies have captured insights in relation to PA and PL within the context of EY education, making this research a valuable contribution to the existing literature as well as providing considerations for PA and PL benchmarking and potential intervention design and delivery. The findings from this research hold potential for dissemination through a variety of practice-based networks, including early childhood education sharing platforms such as Physical Education Matters and Nursery World, as well as PA and obesity working groups such as the Derby children’s healthy weight system network. Further, this study highlights a crucial gap between knowledge of PA recommendations and a PL-informed approach in the EY, which would be of value to community, training, and collaborative EY stakeholders such as the Active Partnerships Early Years working group, the National Early Years Active Start Partnership (NEYASP), the Childrens Alliance, the Health, Exercise and Nutrition for the Really Young (HENRY) programmes as well as academic and research partners interested in the need for a more comprehensive understanding and system-wide approach towards integrating PA and the concept of PL into the EYFS.

## 4. Conclusions

It is evident that PA recommendations for under 5-year-olds and the concept of PL are not ‘reaching’ early education effectively, and some considerations need to be made (e.g., messaging, policy, and research) to better support EY stakeholders and educators to more effectively facilitate children’s PA and develop PL within this critical age group. Whilst PA and PL are considered to play a fundamental role in preschoolers’ development (with significant physical, behavioural, and health benefits), variations in understanding, terminology confusion, and limited good practice examples pose challenges. Additionally, environmental factors, training, and policy constraints shape how effectively PA guidelines are applied. Finally, the importance of a holistic approach to interventions, stakeholder involvement, and the role of evidence-based practices emerged as key factors for supporting a PL-informed approach in the EYFS. The positive reception of the PL-EY model and potential usefulness of benchmarking PA and PL suggests a promising direction for future resources, education, and intervention development to nurture PA and PL at the earliest opportunity.

## Figures and Tables

**Table 1:** Summary of themes and key quotes.

Theme	Sub-Theme	Quote
1. Importance of PA & PL	1.1. Significance for Development	“Physical activity and movement are fundamental to everything else. They are crucial and fundamental to every child’s development”. (EE1)
		“You can kind of hit the top iceberg if you want to by just introducing the term, or you can get right down to the very bottom of that iceberg and you can really start to explore the real benefits of what physical literacy is”. (EE2) “Allow natural physical play to take place…that are safe but obviously have got lots of risks and challenges”. (F1O1) “Families are complaining about the children’s behaviour and saying that…they’re being destructive at home and they can’t control their behaviour—and we explore what happens if you go and give them an opportunity to be active”. (F4O3)
	1.2. Physical, Behavioural & Health Benefits	“It’s just what we are born to do. Fundamentally, our bodies are designed to move and the sedentary lifestyle that we live in does not work well with our bodies and brains, and that’s why we’re seeing an increase in mental health behaviours.” (EE3)
		“The more active… you know, they get the heart rate up but also they’re learning how to climb and learning how to socialise and take turns.” (EM11) “Movement through play for fine and gross motor skill development”. (F4O2) “We saw the improvement afterwards and it was huge for behaviour, it was huge for communication, especially within the girls and preschool…it benefitted them educationally”. (F3E1)
2. Knowledge about PA & PL	2.1. Variation of Understanding	“There is almost this belief that with children between 0–5 it just happens naturally, so we don’t need to do anything—you open the door and let them run and that’s it.” (EE1)
		“When you throw physical education into the into the mix with this, that’s where I think a lot of the barriers come because there’s lots of teachers that feel like they aren’t qualified in being able to deliver PE”. (EE2)
		“The good thing is it’s supporting them at the earliest stage [to understand PL]…the negative is that it can possibly come across as overcomplicating something.” (EM11)
		“In a nursery setting, when the majority of the workers have like their Level 3 in childcare, they don’t really cover the sort of PL side and I don’t think they’d actually know the PA requirement”. (F2PE1)
	2.2. Terminology Confusion	“There are so many interchangeable terms that I think there needs to be something for educators to really comprehend and understand the PD process”. (EE3)
		“Consistency of language… finding a very clear message will be beneficial.” (EM07)
		“Does the phrase physical literacy itself act as a potential barrier?… that it sounds academic and it sounds difficult.” (ED10)
		“It’s the understanding of the physicality of actions and the competence or developing the competence”. (F1M1)
		“Physical literacy, when you say that word, it’s like ohh. It’s gotta be in relation to reading”. (EM11)
3. Barriers to Implementing PA Guidelines	3.1. Resource Limitations	“There’s no talk about physical literacy in early years. There’s no policy. There’s no documentation, and there’s nothing that says anything about what physical activity actually looks like in early years.” (EE2)
		“[important that we] have resources that educate and support professionals and parents and caregivers on the link between physical literacy and physical activity so that they can understand”. (EM11)
		“Practitioners get bombarded with posts about everything, like safeguarding and anything else like additional needs and stuff like that”. (EE4)
	3.2. Policy & Curriculum	“We need to value that [PA and PL] and have a whole team approach and a whole school approach”. (F1E1)
		“Everybody says our curriculum just does not allow time.” (EE3)
		“Getting the Department of Health and Department for Education to talk about anything together is challenging.” (ED10)
		“[a need] for there to be kind of a leading voice about physical activity in the early years”. (EM07)
		“The EYFS framework and school policies formalising the EY has had a significant role in reducing play experiences in the EY classroom”. (P121)
	3.3. External Factors	“Parents were either very supportive or had very different views on what was appropriate for their children.” (EE1)
		“Lack of understanding of the value of PA by senior leaders”. (P5)
		“The other barrier I see is a lot of practitioners, and especially the older ones, don’t do much physical activity themselves.” (EM11)
		“Focus tends not to be on the physical activity. It tends to be on speech and language and things like that. I think the way they traditionally did deliver doesn’t include physical activity. They don’t think about doing the two together.” (EM11)
		“The education side needs to recognise that the evidence being presented by the health side is not a pretty picture and needs to be actively addressed.” (ED10)
		“The challenge is that [settings] are all different, so some don’t have outside space, some have a very small outside space.” (EM11)
		“They’re not sat at tables yet…and are able to explore their own physicality in a completely different way to an older child”. (F2M1)
4. Determinants for Facilitating PA/PL	4.1. Role of Environment	“It depends on the environment, the people around them, the situation that that child is in, whether they can achieve it [PA recommendations] or not.” (EE2)
		“If it’s an environment where it’s small world play and children are sitting on a mat or looking at circle time, then that’s going to be very different levels of physical activity, mainly sedentary behaviour. So the environment will dictate that.” (EE3)
		“Planning your curriculum to ensure there are those key moments in the day for physical activity to happen.” (EE1)
		“Every setting is so different…some settings do discrete PE sessions and some have a real emphasis on everyone moving…” (EM07)
	4.2. Training and Support	“Need to have access to reliable PL research and resources with a practical application to EY and it needs to be embedded into all EY training”. (P27)
		“The senior leadership team need to tick this box—make sure you’re doing a gross motor run around outside for a little bit, fine motor give them a couple of bits of play dough and then job done”. (EE3)
		“Notwithstanding the fact that the policy isn’t gonna change from the top, it may well be fertile ground to work with individual setting managers and their staff teams.” (ED10)
		“…to provide top-quality training for our educators, whether they’re childminders or volunteers at a playgroup or educators within a setting.” (EM07)
		“I don’t think I’ve really worked with any setting either myself or through some of the programmes where I’ve met a setting that has not improved their understanding. That can implement some change.” (EM11)
	4.3. Role of the Educator	“You have to be like the facilitators for the children to be able to hit those [PA] guidelines”. (EE2)
		“The educators will either facilitate and enable or restrict.” (EE3)
		“If the staff go out in the garden and just lean against a tree or lean against a fence and they are sedentary, what are the children going to learn?” (EE4)
		“If you’re a practitioner that doesn’t actually do any physical activity, it’s really hard for them to understand what that means. A role model is probably one of the most crucial things you can ever have within a setting”. (EE1)
		“Responsive adults to model and support movement. Allow it to be child-led—adults to follow the child’s lead”. (P118)
5. Designing Interventions	5.1. Holistic Approach	“Every time that we have an intervention… the case for change is that the status quo isn’t an option.” (EE5)
		“We need to break that chain of behaviours and perspectives.” (EE3)
		“Physical literacy can’t be delivered. All we want is physical literacy informed practice. That’s what we need to go after.” (EE5)
		“Understanding the absolute necessity and importance of understanding physical activity in all aspects”. (EE1)
		“We need to get back to basics. Really big issues, they’re things that could have been solved if they had more opportunity for that freedom to move”. (F1E1)
	5.2. Involvement of Stakeholders	“It has to come from the setting and the leader of the setting to help parents in a drip-fed way understand why it’s important”. (EE1)
		“The good thing is it’s supporting them at the earliest stage [to understand PL]”. (EM11)
		“There was a period of time… when it was an active part of the sessions that I used to run… inspectors had commented that outdoors… physical activity was a chance for the children to not be learning”. (ED10)
	5.3. Evidence-Based Practices	“We just keep being pushed back all the time in terms of ‘What’s your evidence?’ I think any type of data strengthens that… it becomes helpful because you’ve got something concrete.” (EE5)
		“[We need] evidence-based research to say how important this is.” (EE1)
6. PL-EY Model, Measurement & Profiling	6.1. Reception	“It’s important that we benchmark ideas of how many children accessing PA are having their required 180 min of physical activity and where they are in their PL journey so we can take positive action for those children”. (EM07)
		“I think the way the model has been put together is non-threatening and cleverly done.” (EE1)
		“[PL-EY model] would be useful, but I think it needs to be contextualised in the discussion”. (ED10)
		“Equated it [PL] with motor skills, but now I see how the other elements seem to come into it”. (F2P1)
	6.2. Challenges in Measurement	“You can’t ask the five-year-old whether you’ve been doing 3 h of physical activity a day, and so I think that that’s a challenge”. (EM11)
		“The amount of time a child is active across their day is so hard to determine”. (EE2)
		“It’s very difficult to kind of have the holistic view of a child sometimes in that snapshot data”. (EE4)
		“Schools are already measuring a lot of things, so it would need to make sure that the administration burden of monitoring PL wouldn’t be too onerous”. (P76)
		“Measuring and quantifying is the hard part”. (EE1)
		“PL has many components, and I think it is difficult to measure all of them accurately/reliably in this age group”. (P46)
	6.3. Importance of Data	“Data talks, doesn’t it really?… We are supposed to be in an environment where policy is based on data and what works.” (ED10)
		“Unless there’s some evidence, it’s very hard to make anything change.” (EE1)
		“We need to make sure that assessments are relevant and supporting what they do next when it comes to physical activity.” (EE1)
		“Early years practitioners being really well skilled in observing the children and are experienced at making benchmarking judgements”. (EM07)
		“You might not be able to measure all of these things, but with PL if you can go for the things that are key it is feasible and sort of conceivable that you will get a reliable measurement of the concept that you are trying to get at”. (EA6)
		“I think if you identify certain profiles you can target skills… Or opportunities”. (EA6)

**Table 2:** PD outcomes ranked in order of importance.

Ranking Order	Outcome	Mean Ranking ^1^
1	Enjoyment & Motivation	3.36
2	Gross Motor Skills	3.62
3	Emotional Development	4.34
4	Fine Motor Skills	4.77
5	Social Development	4.79
6	Fundamental Movement Skills	5.39
7	Achieving enough PA	5.56
8	Strength Development	6.93
9	Understanding & Knowledge	7.92
10	Cardiovascular Fitness	8.32

^1^ Kendall’s W calculation conducted using SPSS.

## Data Availability

The data presented in this study are available upon request from the corresponding author. The data are not publicly available due to ethical and GDPR reasons.

## Appendix A

**Table A1:** Qualitative Participant Coding & Role.

Participant Code	Focus Group or Interview ^1^	Role
F2PE1	F2	A Parent, Practitioner & Activity Provider
F1M1	F1	Practitioner
F1E2	F1	Practitioner
F4PE1	F4	A Parent & Practitioner
F2P1	F2	A Parent
F4PO1	F4	A Parent & Other (charity worker)
F1O1	F1	Other (community organisation)
F1H1	F1	Headteacher (Infant School)
F1E1	F1	Teacher trainer
F4O2	F4	Other (community organisation)
F3E1	F3	Practitioner & Other (sports coach)
F3P1	F3	A Parent
F4P1	F4	A Parent
F4O3	F4	Activity Provider
F2M1	F2	Assistant Head EYFS & KS1
F1O1	F2	Activity Provider
F3E2	F3	Practitioner
F4PEO1	F4	A Parent, Practitioner & Activity Provider
F3E3	F3	Practitioner
F3E4	F3	Practitioner
F3E5	F3	Practitioner
F4O4	F4	Other (community organisation)
EE1	I1	Educator (trainer)
EE2	I2	EY PE Coach
EE3	I3	Practitioner (trainer and direct delivery)
EE4	I4	Practitioner (direct delivery)
EE5	I5	Practitioner (trainer)
EA6	I6	Academic
EMO7	I7	Programme Manager (charity)
EEM8	I8	Area Manager (private nursery chain)
EEM9	I9	Nursery Manager
ED10	I10	Director (EY membership organisation)
EM11	I11	Programme Manager (charity)

^1^ F = Focus Group, I = Interview.

## Appendix B

### Appendix B.1. Expert Interview Script

Introduction and Background

- Could you please introduce yourself and provide a brief overview of your expertise and experience in EY education?
- What motivated you to pursue your work for or in relation to EY education?

Theme 1: Exploring knowledge and beliefs

- Where do you perceive PA experiences to sit in the context of EY education? Do you view PA as PE, or active play? Do you see it as a broader concept encompassing various movement experiences beyond structured lessons? Please elaborate on your viewpoint.
- What do you consider ‘play’ to be? How do play and PA interlink?
- What is the first thing you think about when I say “PL”?
- Do you think there is any relationship between PA/movement behaviours and PL?

Theme 2: Understanding and perceptions of PA and PL in practice

- Are you aware of the recommended PA guidelines for under 5s?
- Do you believe the PA government guidelines of 180 min per day for children aged 5 years and below are sufficient and achievable? What barriers do you think teachers and schools face to achieving at least 30 min/day of MVPA for children?
- Do you feel there is adequate opportunity for children in the EYFS to reach these recommendations?
- How well do you understand the concept of PL in the context of EY education?

If well, why do you think that is?
If not well, why do you think that is?

- Could you share any trends or developments you’ve observed in recent years related to PA and PL in relation to the EY?
- Could you share an example from your own work or projects that illustrates the significance of being more active and/or PL?

Theme 3: Influencing PA/PL in the EY

- In your opinion, what needs to be thought about more, adapted, or improved to enhance PA and PL?
- How much do you think about PL and PA together in the work you do?

Theme 4: Designing Interventions

- What do you believe are the key themes or factors that need to be addressed when designing interventions to promote PA and PL among preschool children?
- What challenges or barriers do you foresee in designing and implementing interventions for promoting PA and PL in preschool children?
- How can these challenges be addressed to ensure the successful implementation of such interventions?
- Reflecting on your experience, what would you consider as the strengths in promoting PA and PL among preschool children?
- On the other hand, can you identify any weaknesses or limitations that might impact the effectiveness of these efforts?
- From your perspective, can you identify any specific opportunities or potential areas for improvement in promoting PA and PL in the preschool age group?
- How can these opportunities be leveraged to create innovative and impactful interventions?

Theme 5: Feedback on the PL-EY model
Show PL-EY model/poster

- When considering the PL in EY (PL-EY) Model, how valuable do you think it would be in educating stakeholders about the relationship between PA and PL?
- How might this model influence your approach to promoting PA and PL among preschool children?
- Can you share your thoughts on the potential benefits and advantages of teaching and sharing a PL-EY model in the context of EY learning? Are there any disadvantages you envisage?

Theme 6: Feasibility of PA/PL Measurement and Profiling

- Do you believe it is important to objectively measure PA levels and PL in preschool children?

If yes, why?
If not, why?

- What are your thoughts on the feasibility and practicality of implementing a measurement and profiling approach for PA and PL in the EY age group?
- Can you share your views on the potential benefits and challenges associated with measuring and profiling PA and PL in preschool children?
- If data on the movement behaviours and PL levels were captured and enabled the creation of profiles that you could see and respond to, how do you think this might support how you teach/develop/encourage children to be active?
- Would you feel confident delivering an intervention that potentially benchmarks PA and PL levels if training/tools like accelerometer/questionnaires were provided? Why?
- Can you share a case study or example of a situation where interventions promoting PA and PL have led to positive changes? What contributed to the success in this case?
- Or a case study of someone who has changed for the better?

Closing:

- Do you have any other thoughts or views you would like to share?
- Can you tell me why you decided to participate in this interview?
- What has it felt like to participate in this interview? Is it what you expected? (If not, what did you expect?)

### Appendix B.2. Focus Group Script

Theme 1: Exploring knowledge & beliefs

- What comes to your mind when you hear the words “PA” in children aged 5 years and under?
- Where do you perceive PA to sit in the context of EY education? Do you view PA as PE, or active play? Do you see it as a broader concept encompassing various movement experiences beyond structured PE lessons? Please elaborate on your viewpoint.
- What do you consider ‘play’ to be? How does play and PA interlink?
- What is the first thing you think about when I say “PL”?
- Do you think there is any relationship between PA/movement behaviours and PL?
- What do you feel children achieve from being physically active?
- Do you notice any key differences between children who are more physically capable and others? Physically, socially, academically?
- Who or what do you think are the key sources or influencers of PA and PL in children of this age group?

Theme 2: Stakeholders’ understanding and perceptions of PA and PL in practice

- Are you aware of the recommended PA guidelines for under 5s?
- Do you believe the PA government guidelines of 180 min per day for children aged 5 years and below are sufficient and achievable? What barriers do you think teachers and schools face to achieving at least 30 min/day of MVPA for children?
- Do you feel you provide adequate opportunity for children to reach these recommendations?
- How well do you understand the concept of PL in the context of EY education?
- If well, why do you think that is?
- If not well, why do you think that is?
- Do you think the service you provide provides adequate opportunity for children to develop their PL?

If yes, why?
If no, why not?

- How much do you think about PL and PA together?

What are your thoughts on the role of: parents, headteachers, coaches/activity providers,
other?
Theme 3: Influencing PA/PL in the EY

- What equipment and facilities are available in the nursery to facilitate PA and PL, both indoors and outdoors?
- Do you consider indoor or outdoor space more important for promoting PA and PL in children, or do you see both as equally important?
- Are there any existing projects or initiatives in place at the nursery to promote PA and PL?
- In your opinion, how can the environment and facilities be further adapted or improved to enhance PA and PL?

Theme 4: Designing interventions

- What do you believe are the key themes or factors that need to be addressed when designing interventions to promote PA and PL among preschool children?
- What challenges or barriers do you foresee in designing and implementing interventions for promoting PA and PL in preschool children?
- What would you say are the strengths in your environment/setting/teaching, etc.?
- Are there any weaknesses?
- Can you identify any specific opportunities or potential areas for improvement in promoting PA and PL in this age group?

Theme 5: Feedback on the PL-EY model

- Show the PL-EY model—how valuable do you think a PL in EY (PL-EY) Model would be in educating stakeholders about the relationship between PA and PL?
- How might this model influence your approach?
- Can you share your thoughts on the potential benefits or advantages of teaching and sharing a PL in EY (PL-EY) Model in promoting PA and PL among preschool children?
- Are there any disadvantages you envisage?

Theme 6: Feasibility of PA/PL Measurement and Profiling

- Do you believe it is important to objectively measure PA levels and PL in preschool children? Please explain your answer.
- What are your thoughts on the feasibility and practicality of implementing a measurement and profiling approach for PA and PL in the EY age group?
- Can you share your views on the potential benefits and challenges associated with measuring and profiling PA and PL in preschool children?
- If data on the movement behaviours and PL levels were captured and enabled the creation of profiles that you could see and respond to, how do you think this might support how you teach/develop/encourage children to be active?
- Would you feel confident delivering an intervention that potentially benchmarks PA and PL levels if training/tools like accelerometer/questionnaires were provided? Why?
- Can you tell me about something that works and why? Or a case study of someone who has changed for the better?

Closing:

- Do you have any other thoughts or views you would like to share?
- Can you tell me why you decided to participate in this focus group?
- What has it felt like to participate in a focus group? Is it what you expected? (If not, what did you expect?)

### Appendic B.3. Survey Questions (Omiting Consent and Contact Details Questions)

Qu 5. Are you (select any that apply)

- A Parent
- Teacher/Practitioner
- Childminder
- Activity Provider
- Other (please specify)

Qu 6. With which gender do you most identify?

- Male
- Female
- Non-binary/third gender
- Prefer not to say

Qu 7. What is your age?

- <18
- 18–24
- 25–34
- 35–44
- 45–54
- 55+
- Prefer not to say

Qu 8. What is the age of the child/children in your care? You can select all that apply.

- 3
- 4
- 5

Qu 9. What is the environment/education setting type you work within? If you are a parent, answer what early education setting your child attends (if more than 1 applies, select which one you consider to be the main education environment).

- State School (school/nursery school)
- Private, voluntary, or independent nursery setting
- Childminder
- Homecare
- Other (please specify)

Qu 10. Please provide the setting postcode
Qu 11. In relation to the physical development outcomes of 3–5-year-olds—drag and drop the statements to rank which ones you consider to be the most important (1) to least important (10)

- Development of gross motor skills (that involve control of larger muscle groups, e.g., jumping)
- Development of fine motor skills (control of small muscles, e.g., hand, finger, lip & eye movements)
- Fundamental movement skills (skills that involve the combination of various movement patterns, e.g., catching)
- Increased enjoyment & motivation (e.g., pleasure, satisfaction, and fun in movement and play)
- Achieving enough PA (body movements of varying intensities, often gained through play)
- Emotional development (e.g., resilience, empathy, self-identity)
- Social development (e.g., language, communication, relationship-building)
- Understanding & knowledge (e.g., rules, why we are active, the benefits)
- Strength development (e.g., core strength & posture, muscular strength)
- Improving cardiovascular fitness (e.g., heart & lung health, overall stamina, and endurance)

Qu 12. Are you aware that there are recommended daily PA levels for 3–5-year-olds?

- Yes
- No
- Not sure

Qu 13. Please select what you think are the UK Chief Medical Officers’ PA guidelines for 3–5-year-olds

- At least 60 min of moderate-to-vigorous activity per day
- 180 min (to include at least 60 min of moderate-to-vigorous activity) per day
- 120 min per day
- 60 min (to include 30 min of moderate-to-vigorous) per day
- 150 min (to include 75 min vigorous activity) per week
- Not sure

Qu 14. The guidelines are 180 min with at least 60 min/day of moderate-to-vigorous activity. Do you feel there is adequate opportunity for children/your child aged 3–5 years to reach these recommendations?

- Always
- Often
- Sometimes
- Rarely
- Never

Qu 15. Please explain your answer
Qu 16. What do you see as barriers in 3–5-year-olds reaching the recommended levels of PA?
Qu 17. How familiar are you with the term ‘P’

- Not familiar at all
- Slightly familiar
- Moderately familiar
- Very familiar
- Extremely familiar

Qu 18. PL is our relationship with movement and PA throughout life. How we move (physical), connect (social), think (cognitive), and feel (affective) play an equal crucial role in shaping our PL
Please consider the PL in the EY (PL-EY) poster below. How useful do you think it is in educating you about PL in relation to 3–5-year-olds?

- Extremely useful
- Very useful
- Moderately useful
- Slightly useful
- Not at all useful

Qu 19. How might this PL-EY model influence your approach to PA and PL among 3–5-year-old children?
Qu 20. There are 3 ‘prime areas’ of learning and development in the EY Education Curriculum. In which development area do you think PL should sit?

- Physical Development
- Personal, Social, and Emotional Development
- Communication and Language
- None of the 3 prime areas listed above: Life curriculum—an overarching concept (not limited to a single development area but which influences and is influenced by them all)

Qu 21. In your opinion, what needs to be thought about more, adapted, or improved to enhance PL for 3–5-year-olds?
Qu 22. How important do you think PL is in relation to 3–5-year-olds?
Select 1–10
Qu 23. We would like to get your views on the feasibility of measuring and profiling the PA and PL levels of 3–5-year-olds.
Would information on PL and PA levels influence your approach to the development of 3–5-year-old child/ren?

- Definitely yes
- Probably yes
- Maybe
- Probably not
- Definitely not

Qu 24. What do you believe are the key factors that need to be considered when designing interventions to promote PA and PL among 3–5-year-old children?
Qu 25. Do you think it would be useful to measure and profile PA levels in 3–5-year-olds?

- Definitely yes
- Probably yes
- Maybe
- Probably not
- Definitely not

Qu 26. Do you think it would be useful to measure and profile PL levels in 3–5-year-olds?

- Definitely yes
- Probably yes
- Maybe
- Probably not
- Definitely not

Qu 27. If you have any concerns about measuring and profiling PA and PL levels in 3–5-year-olds, please outline them below.
Qu 28. Do you have any additional recommendations for support, such as resources, equipment, or training, that would assist you in promoting PL and offering positive movement opportunities in your role with 3–5-year-olds?
Qu 29. If there are any other comments you would like to make, please add them here. Thank you.
